# Knowledge, attitudes, and behavioural intentions toward IPTp-SP use among pregnant women in rural Nigeria: A theory of reasoned action approach

**DOI:** 10.5281/zenodo.21644673

**Published:** 2026-07-28

**Authors:** Patricia Ogba, Tunde Alabi, Andrea Baumann, Norm Archer, Joshua Eniojukan, Bonny Ibhawoh, Deborah DiLiberto

**Affiliations:** 1 Global Health Office, Faculty of Health Sciences, McMaster University, Main St. W, Hamilton, Ontario, Canada.; 2 Department of Sociology, University of Lagos, Akoka, Nigeria.; 3 Degroote School of Business, McMaster University, Main St. W, Hamilton, Ontario, Canada.; 4 Department of Clinical Pharmacy, Niger-Delta University, Wilberforce Island, Bayelsa, Nigeria.; 5 Department of History, McMaster University, Main St. W, Hamilton, Ontario, Canada.

## Abstract

**Background:**

Globally, malaria cases increased from 228 million in 2018 to 282 million in 2024, with the African region accounting for 95% of the cases. Nigeria bears the highest malaria burden in the world, and pregnant women carry a significant number of the cases. Malaria in pregnancy (MiP) may have negative implications for maternal and neonatal health. The WHO-recommended intermittent preventive treatment using sulphadoxine-pyrimethamine (IPTp-SP) helps to prevent MiP, but its uptake is low in Nigeria. Using the theory of reasoned action (TRA), this study investigated the attitudes and intentions of pregnant women in Bayelsa State towards the use of IPTp-SP.

**Methods:**

The study adopted qualitative methods, including focus group discussions with pregnant women and key informant interviews with relevant community stakeholders. Six focus group discussions and seventeen key informant interviews were conducted with pregnant women and stakeholders, respectively. Data were managed using NVivo (version 12) and analysed thematically and deductively in line with the TRA.

**Results:**

Pregnant women had limited knowledge of preventing MiP and specifically IPTp-SP use. This resulted in a negative attitude towards the preventive technique. Interestingly, while the role of significant others, such as traditional birth attendants (TBAs), husbands and mothers-in-law, was acknowledged, many pregnant women did not succumb to the social pressure to use IPTp-SP when they did not think it was best for them. Many women intended to use IPTp-SP but had not done so at the time of the study due to structural barriers, poor access to facilities, and the unavailability of IPTp-SP.

**Conclusions:**

The use of IPTp-SP is poor among pregnant women in the sample. These women have their own will and are not necessarily pressured by social norms. Intentions may not translate to actual practice or use if structural barriers are not eliminated.

## Introduction

Globally, there is evidence that the prevalence of malaria has increased in the last few years. According to the 2019 World Malaria Report of the World Health Organisation (WHO), there were about 228 million malaria cases worldwide, and 95% of these were in Africa [1]. However, the 2024 and 2025 World Malaria Reports show that the number of cases had increased to about 263 and 282 million [2,3], respectively, with Nigeria carrying the heaviest burden of 24.3%, followed by the Democratic Republic of Congo (12.5%), Uganda (4.7%), Ethiopia (4.4%), and Mozambique (3.6%) [3]. By implication, Nigeria's malaria burden is greater than the combined burdens of the second-, third-, and fourth-largest carriers. Pregnant women are particularly vulnerable to malaria due to the reduced immunity associated with pregnancy [4–7].

In 2023, according to WHO [2], 33 moderate-to-high transmission countries in the WHO African Region recorded an estimated 36 million pregnancies, out of which 12.4 million (34%) were infected with malaria. This indicates that malaria in pregnancy (MiP) remains a significant public health challenge in malaria-endemic regions, particularly in Africa, where transmission is intense and persistent [2,4,8]. MiP is associated with a range of adverse maternal and neonatal outcomes, including maternal anaemia, preterm delivery, low birth weight, stillbirth, and increased risk of neonatal mortality [2,4,8–11]. It is estimated that MiP contributes up to 200,000 infant deaths annually in Africa and remains a major contributor to maternal morbidity and mortality [10,12]. Nigeria is one of the two countries where pregnant women have the highest exposure to malaria infection, and an estimated 11% of maternal deaths are due to malaria [13].

To avert the consequences of MiP, WHO recommends, in combination with other interventions, the use of intermittent preventive treatment with sulfadoxine-pyrimethamine (IPTp-SP) under directly observed therapy (DOT), as part of antenatal care (ANC) in malaria-endemic areas [2,14]. IPTp-SP entails administering at least three doses of sulfadoxine-pyrimethamine (SP) to women during pregnancy, with each dose administered monthly on ANC visits starting in the early stage of the second trimester until the time of delivery [4,11,14,15].

In Nigeria, IPTp-SP, also known as Fansidar, is included as part of the national malaria treatment protocol and offered free of charge during ANC visits under the DOT strategy [4,14]. However, uptake remains suboptimal due to systemic, sociocultural, and individual-level factors [6,9,16]. Evidence shows that ANC utilisation in Nigeria remains low [12], particularly in rural areas, and misconceptions about the safety and efficacy of IPTp-SP persist among pregnant women and community stakeholders [9].

Some critical characteristics of Nigeria, relevant to public health, include its diversity and notable differences in sociocultural and health-seeking behaviours across states and regions [17,18]. Hence, some states or areas are more vulnerable to malaria and are more likely to engage in preventative behaviours than others. Hence, this study examined the knowledge, attitudes, and behavioural intentions of pregnant women in two rural communities in Bayelsa State, Nigeria, regarding the use of IPTp-SP. Bayelsa State is a riverine area, which may increase vulnerability to MiP [19–22]. Additionally, over half of the pregnant women in this State do not visit the ANC [22] where they can access IPTp-SP.

Given the multifactorial determinants of IPTp-SP uptake, this study specifically examined the psychosocial determinants of IPTp-SP use among pregnant women using the Theory of Reasoned Action (TRA) framework, while recognising that ANC attendance is an important upstream determinant warranting separate investigation. The TRA framework is suitable for examining how individual knowledge, attitudes, and perceived social norms influence behavioural intentions and health-related actions, such as IPTp-SP use. By identifying the key facilitators and barriers to IPTp-SP uptake, this research aims to inform the development of culturally relevant, context-specific interventions to improve MiP outcomes in resource-constrained settings.

### Theoretical framework

The TRA was developed by social psychologists Martin Fishbein and Icek Ajzen in 1980. It is a psychological model that predicts and explains relationships between attitudes, intentions, and behaviours across various contexts [24,25]. The TRA proposes that an individual’s choice to adopt a particular behaviour is primarily influenced by their intention to perform that behaviour. This intention is shaped by two core constructs: attitude toward the behaviour and the subjective norms surrounding it [24,26]. Attitudes are shaped by beliefs about the expected outcomes of a behaviour and the value placed on those outcomes, whereas subjective norms refer to others' perceived expectations and the motivation to comply with them [24–26]. This theory suggests that individuals are more inclined to engage in a behaviour when they hold a positive attitude towards it and perceive that significant others approve of their decision to do so.

The TRA was selected because the primary aim of this study was to understand how pregnant women's knowledge, attitudes, perceived social influences, and behavioural intentions shape IPTp-SP uptake, rather than to develop a comprehensive model encompassing structural and health-system determinants. Although other behavioural theories incorporate additional constructs such as self-effcacy and environmental barriers, TRA provides a suitable framework for examining how personal beliefs and subjective norms interact to influence behavioural intentions. In this context, the TRA stipulates that pregnant women’s behaviour regarding the uptake of IPTp-SP is impacted by their intention to use the drug. This intention is influenced by their attitudes towards IPTp-SP use and the subjective norms surrounding it. Attitude towards IPTp-SP use stems from pregnant women’s beliefs in IPTp-SP’s benefits, efficacy, and safety. In the context of this study, subjective norms refer to the influence of significant others, such as religious leaders, spouses, and mothers-in-law, whose approval or disapproval can influence a pregnant woman’s adherence to IPTp-SP recommendations.

Understanding these psychosocial determinants is crucial for designing culturally sensitive interventions that can enhance adherence to malaria prevention strategies during pregnancy.

## Methods

This article draws on a broader exploratory, descriptive qualitative study investigating community-level contextual factors influencing the uptake of preventive treatments for malaria during pregnancy. That study was conducted in Yenaka and Sabagreia, two rural communities in Bayelsa State, Nigeria. The region's swampy, waterlogged terrain enables ideal conditions for year-round mosquito breeding, sustaining high levels of malaria transmission [19,22,27]. Malaria remains a significant cause of mortality, worsened by limited access to formal healthcare, widespread reliance on traditional remedies, and self-medication driven by poverty and the area’s challenging geography [19,20,22,27]. Yenaka (designated BC1) has only one health centre, which is partially functional and lacks adequate equipment, essential drugs, and emergency facilities [28]. Sabagreia (BC2) is served by two poorly equipped cottage hospitals operated by community health extension workers. Neither facility operates round-the-clock.

The main study involved pregnant women and key stakeholders, including spouses, mothers-in-law, religious leaders, community leaders, and traditional birth attendants (TBAs). All participants were approached face-to-face and recruited with the assistance of four trained research assistants. Pregnant women were conveniently sampled and recruited directly from TBA clinics in the two communities, and their partners were also invited to participate. Stakeholders were purposefully selected for their influential roles in the community. This sampling approach captured both the lived experiences of pregnant women and the perspectives of those who can influence pregnant women’s health-seeking behaviours. A total of 53 participants took part in this study.

In all, six focus group discussions (FGDs), three in each of the two communities, comprising six pregnant women each, were conducted. Seventeen stakeholder interviews were conducted with community leaders (2), TBAs (3), religious leaders (4), spouses (4) and mothers-in-law (4).

Participants’ age ranged from 18 to 38 years. Of the 36 pregnant women, 50% had no formal education, 35% completed primary school, and 15% reached secondary education. About 42% were married, while 58% were unmarried. Though residing in the same communities, they represented various ethnic groups, including Eppie, Atisa, Ijaw, Isoko, Ikorobasi, Akwa-Ibom, Kolokuma, and Ikot Ekpene. All identified as Christians, with most (96%) working as farmers and 4% as housewives.

For data collection, we adapted Nyaaba et al.'s interview guide [6] and piloted it with 10 pregnant women from two neighbouring communities; their responses were excluded from the main study. The pilot confirmed the questions were precise and culturally appropriate, requiring only minor language adjustments. The pregnant women preferred pidgin English (colloquial English), so the focus groups were conducted in this language. Before each interview, stakeholders chose their preferred language: spouses, TBAs, and mothers-in-law opted for Ijaw, while religious and community leaders preferred English.

The interview guide was developed in English. Because suitable professional translators were not available for the local dialects spoken in the study communities, the guide was not formally translated. Instead, trained bilingual research assistants, who were indigenous to the study communities and fluent in both English and the local dialects, verbally interpreted the interview questions into participants' preferred dialects during the in-depth interviews (IDIs) and FGDs, as needed. The same research assistants subsequently transcribed and translated the interviews into English for analysis. Prior to data collection, the research assistants were trained on the interview guide to promote consistent interpretation of the questions across interviews. The guide included open-ended questions to measure participants' attitudes towards IPTp-SP uptake behaviour, normative beliefs and subjective norms regarding IPTp-SP use, as well as behavioural intention and actual behaviour of using IPTp-SP.

### Data collection procedures

We (P.O. and four trained research assistants) collected data through FGDs and IDIs between 9 May 9 and 29 June, 2022. FGDs were conducted with pregnant women to explore shared experiences, social norms, and collective perceptions influencing IPTp-SP uptake. This approach was chosen because IPTp-SP-related decisions are often shaped by interpersonal and community-level influences, which are well captured through group interaction. FGDs also allowed participants to reflect on and build upon each other’s experiences. However, potential limitations include social desirability bias, a few participants dominating the conversation, and reduced disclosure of sensitive experiences, which were considered during facilitation. The FGDs were conducted in community halls, and the IDIs were conducted in stakeholders' homes and offices. This research involved 17 IDIs with stakeholders and 6 FGDs, each comprising 6 pregnant women, for a total of 36. Interviews with stakeholders lasted 30-45 minutes, and FGDs with pregnant women lasted 1-1.5 hours. Conducted in English, pidgin English, and Ijaw, the interviews continued until we reached data saturation.

### Data analysis

The digitally recorded interviews in Ijaw and Pidgin English were interpreted and transcribed directly into English by two native speakers, as transcriptionists fluent in Ijaw were not available. The interview transcripts were managed and analysed using NVivo 12 qualitative analysis software by QSR International.

The analysis was conducted using a deductive coding framework informed by the TRA. In line with the TRA, two team members (P.O. and T.A.) coded and organised the study results into participants' attitudes towards IPTp-SP uptake; normative beliefs and subjective norms regarding IPTp-SP use; behavioural intention; and actual behaviour of using IPTp-SP.

### Position statement

The lead author, a Nigerian researcher, completed her secondary and undergraduate education in Nigeria. As a registered nurse and midwife with over nine years of clinical experience in Nigerian hospitals, she is well-informed about how malaria affects pregnant women and their unborn babies. This professional experience informed and motivated her interest in this study. She is also trained in qualitative research methods, skills she acquired during her MSc and PhD studies.

The author engaged in ongoing reflexivity to mitigate potential bias stemming from her background in healthcare. Member checking was conducted after data analysis by sharing a summary of the study findings with participants through the research assistants. For community and religious leaders who could communicate in English, the findings were shared directly by the research team. For pregnant women, partners, TBAs, and mothers-in-law, bilingual research assistants explained the findings in the participants' preferred local dialects. While the insider status of the research assistants who conducted the interviews and transcribed the resultant data fostered the participants’ trust, it required careful reflexive monitoring. Regular debriefings with the assistants were held to examine how their position might influence data collection and interpretation, helping to ensure the findings remained rooted in participants’ lived experiences.

### Trustworthiness and rigour

Trustworthiness in qualitative research, encompassing credibility, dependability, confirmability, and transferability, ensures research quality [29,30]. This study provided credibility through member checking with selected participants from each stakeholder group, due to logistical constraints, and through triangulation. We addressed transferability by situating findings within the existing literature. Further, a community member cross-checked audio recordings with interview transcripts, and the codes developed for analysis by P.O. were validated by a co-author, T.A., adding another layer of quality assurance.

### Ethical considerations

Ethical approval was sought from Canada, the Hamilton Integrated Research Ethics Board [HiREB#: 14382], and Nigeria, Bayelsa State Ministry of Health, with approval number BSHREC/ Vol. 1/21/12/001. Additionally, letters of permission were obtained from the leaders of the studied communities. Stringent ethical measures, including informed consent and confidentiality, were followed, allowing participants to opt out at any stage.

Informed consent was obtained from all participants before their involvement in the study. For participants without formal education, research assistants translated and explained the consent form, originally written in English, into the local dialects. Those who agreed to participate indicated their consent by providing thumbprints. Participants with formal education independently read the consent forms, sought clarifications where necessary, and confirmed consent by signing. All interviews were recorded with permission and securely stored. COVID-19 precautions, including social distancing and mask use, were strictly followed in accordance with Nigeria’s Ministry of Health guidelines.

## Results

Before presenting the themes, it is important to note that this study did not systematically document IPTp-SP uptake. However, participants who discussed Fansidar reported that they were not using it during their current pregnancy because of limited access to antenatal care and the unavailability of the drug.

### Attitude toward the behaviour of IPTp-SP uptake

Participants’ attitudes towards using IPTp-SP for MiP prevention reflected their knowledge and beliefs about MiP and about IPTp-SP’s effectiveness and safety.

#### Participants’ knowledge and beliefs about MiP and IPTp-SP use

Many pregnant women and stakeholders exhibited varied and limited knowledge of MiP and IPTp-SP use. During FDGs, some pregnant women revealed that they were unaware that malaria could affect pregnant women; only a few had this knowledge. For instance, a pregnant woman said:

*“I am not aware that malaria affects a pregnant woman…”* (FGD, BC1).

Another participant noted that:

*“Though I have heard of Fansidar, I had no idea it could protect pregnant women from malaria”* (Mother-in-law, IDI)

Among those who recognised the risk MiP poses, misconceptions about malaria’s causes were widespread. For example, some believed that exposure to sunlight could cause malaria. Furthermore, the majority of pregnant women were unfamiliar with IPTp-SP and did not understand its protective role against MiP. On the other hand, a few stakeholders were familiar with Fansidar and had used it for malaria treatment. However, they were unaware of its role in preventing MiP. Scepticism regarding the drug’s effectiveness and safety was also commonly reported among the study participants:

*“I know about Fansidar for treating malaria, but we are not sure if it really works to prevent it — some people even worry it might not be safe for pregnant women”* (Community Leader, IDI)

In contrast, one community leader demonstrated a more informed perspective, noting that Fansidar benefits pregnant women. In this regard, a participant mentioned that:

*“I have heard of it…I know Fansidar can protect pregnant women from malaria…I did little Pharmacology when I was in school”* (Community leader, IDI)

He was also aware of its appropriate timing and use. He attributed this knowledge to a pharmacology course he took during his undergraduate studies.

Among the TBAs interviewed, all had heard of Fansidar. However, their understanding of its preventive role was inconsistent. While one TBA did not recognise Fansidar as a malaria preventive, the others acknowledged its protective potential but lacked detailed knowledge of its correct dosage and timing during pregnancy:

*“I have heard of Fansidar and I know that they do give it to pregnant women in the clinic”* (TBA, BC2)

*“I know it can help protect against malaria, but I don’t know exactly when or how much to give”* (TBA, BC1)

*“I did not know Fansidar is used to prevent malaria”* (TBA, BC1)

#### Views on prevention and treatment strategies

Pregnant women described a range of strategies to prevent malaria during pregnancy. Most relied on insecticide-treated nets (ITNs), mosquito coils, and sprays to protect themselves against mosquito bites. Some also reported closing doors and windows at dusk to avoid mosquito entry. Stakeholders also noted that many pregnant women use ITNs; although some find these nets uncomfortable and avoid them because they make them feel hot. Other preventive measures mentioned by stakeholders include using insecticides and maintaining clean surroundings to reduce mosquito presence. Although a few women mentioned Fansidar as a preventive method, they reported that they were not using it during their current pregnancy due to limited access to antenatal care and the unavailability of the drug. Others took no preventive measures, largely due to a lack of knowledge on how to protect themselves:

*“Many pregnant women use mosquito nets, but some say they feel too hot and uncomfortable, so they don’t always sleep under them”* (Mother-in-law, IDI)

*“Some of us know about Fansidar to prevent malaria, but we are not taking it this time because the clinic doesn’t have it…"* (FGD, BC1)

*“I don't know anything about how I can protect myself from malaria”* (FGD,BC2)

Regarding malaria treatment-seeking practices, most pregnant women preferred visiting chemists or pharmacies. In contrast, others preferred the services of TBAs, who provided them with herbal concoctions for malaria prevention and treatment. Only a few reported receiving antenatal care at a hospital. The stakeholders also echoed this, attributing the practice to limited access to health centres. Some pregnant women combined both herbal remedies and conventional tablets, depending on which one was readily available.

*“…I usually visit the chemist for my treatment because it is close to me…I don't like to take tablets and injections because they upset my system. I am very scared of injections. These are my reasons for preferring traditional herbs…”* (FGD, BC2)

*“…they treat themselves by taking some native medicine. Honestly, so many of them believe in this native medicine…”* (Religious leader, KII)

*“I take whatever treatment is readily available. If it is a tablet, I will take it; if it is an herbal concoction, I will take it”* (FGD, BC2)

Some pregnant women trusted IPTp-SP and believed it could protect them and their unborn babies from malaria and its adverse consequences, especially if prescribed by a qualified healthcare practitioner (HCP). Others preferred herbal alternatives, citing their fear of tablets and injections, associating them with potential harm in pregnancy. One religious leader elaborated on how rural dwellers, including the educated ones, believe that all malaria-preventing drugs can cause miscarriage:

*“I will take whatever a doctor prescribes… I am scared of all English drugs, including Fansidar”* (FGD BC2)

### Normative beliefs and subjective norms

This refers to the perceived social pressure from significant others regarding the use of IPTp-SP. Normative influences emerged from stakeholder interviews, in which stakeholders described active roles in encouraging pregnant women to attend ANC and follow HCP guidance.

The TBAs in this study generally supported hospital treatment and often referred their clients to health centres, especially when they noticed signs of malaria. One TBA said she usually confirmed with the HCPs at the local health centres whether the client she referred had visited the clinic. While still acknowledging spiritual and traditional beliefs, religious and community leaders encouraged hospital visits and IPTp-SP use as long as qualified HCPs prescribe it. The clergymen asserted that although they believed all sicknesses, including malaria, were from the devil, they told these women to seek hospital care after prayers. Spouses narrated how they supported their wives’ use of IPTp-SP and often provided financial and emotional support. In the same vein, all the mothers-in-law acknowledged the importance of medication and ANC visits, usually offering encouragement based on their experience. Having been pregnant themselves, they know that this state is very stressful for every woman, and the need to be healthy throughout this period is paramount:

*“When I see signs of malaria, I send the woman to the health centre and later check with the nurses to make sure she went”* (TBA, BC1)

*“We believe sickness comes from the devil, but after prayers, we tell pregnant women to go to the hospital and take the medicine the doctors give them”* (Religious leader, IDI)

*“Because I have been pregnant before and I know how important it is for a pregnant woman to stay healthy, I will encourage my daughter-in-law to go to the hospital and to get Fansidar…”* (Mother-in-law, KII)

### Barriers from social influence

Mothers-in-law, TBAs, community leaders, and religious leaders shared instances where some pregnant women, perceived as stubborn, rejected advice to take IPTp-SP or seek prenatal care at health centres. These women often dismissed guidance from elders or caregivers, reflecting a shift away from traditional authority in health decision-making. Some preferred traditional herbs, believing that tablets alone were inadequate:

*“Some pregnant women these days refuse to take the malaria medicine or go to the clinic — they don’t listen to us like before”* (Community leader, IDI)

*“A few of them say the tablets are not enough; they still prefer using herbs instead”* (TBA participant, KII)

### Behavioural intention

Behavioural intention refers to the motivation to use IPTp-SP. Some participants, though not registered in hospitals, expressed willingness to take any drug prescribed by qualified HCPs, trusting their expertise. They further said they were open to using Fansidar if it were available in their communities. For instance:

*“I will take whatever a doctor prescribes…I am happy to take Fansidar as long as it will protect my unborn baby and me from malaria”* (FGD BC2)

Other participants stated that they would avoid IPTp-SP even if offered for free, some citing a general distrust of allopathic drugs. For example, a pregnant woman said:

*“There is nothing that can be done to improve my uptake of Fansidar. I am scared of all English drugs, including Fansidar“* (FGD BC2).

Some pregnant women in this category gave no specific reason for their decision to not take IPTp-SP. The malaria preventive method employed by pregnant women often depended on availability, with some opting for herbal mixtures or Fansidar:

*“My decision to use this drug is based on its availability in the clinic whenever I visit for my ANC…if my mother-in-law introduces a sweet herbal remedy to me when it is not immediately possible for me to visit a hospital and I know it would be effective, then I will use it. But if the opportunity to visit the hospital is immediately available, then I would not take it”* (FGD BC1)

### Behaviour (actual use of IPTp-SP)

This refers to whether the behaviour, IPTp-SP use, was performed. Despite some positive attitudes and supportive social norms, the actual use of IPTp-SP remained low. Although a few pregnant women in this study mentioned Fansidar as a malaria prevention method and stated their awareness of its protective ability, they were not using it in their current pregnancy. They cited limited access to ANC and lack of access to Fansidar as reasons for not using it as a protective measure. Additionally, the prescriptions given in clinics hindered use, as some HCPs still prescribe non-recommended alternatives like Amatem:

*“For this pregnancy, I have not taken Fansidar”* (FGD BC1)

*“The use of fansidar is not common in rural places like ours because it is not readily available and accessible”* (FGD BC2)

## Discussion

Through the TRA lens, this study explored pregnant women’s knowledge, attitudes, and intentions regarding malaria prevention, particularly the uptake of IPTp-SP. The TRA posits that behaviour is guided by behavioural intentions, which are influenced by attitudes toward the behaviour and subjective norms [24,26]. These study findings provide important insights into how these constructs manifest in rural Nigerian communities and contribute to low uptake of IPTp-SP, despite ongoing malaria prevention campaigns.

Participants’ attitudes towards IPTp-SP use were primarily shaped by limited knowledge and widespread misconceptions about MiP and the preventive benefits of IPTp-SP. Although some pregnant women and stakeholders recognised the drug’s protective effect, especially when prescribed by qualified HCPs, many of the study participants remained sceptical about its safety and efficacy. This finding is consistent with previous studies conducted in sub-Saharan Africa, Ghana, and Nigeria [6,31–34], where study participants were unaware of the use and protective benefits of IPTp. However, this finding contradicts previous studies [6,34–37], in which over half of the study participants knew the dangers of MiP, the vulnerability of pregnant women to it, and the use of IPTp-SP for malaria prevention. People’s negative beliefs about IPTp-SP benefits, safety and side effects, and the preference for herbal concoctions influenced their attitudes concerning the outcome of using it, ultimately impacting pregnant women’s use of this malaria prevention tool. This finding is consistent with a previous study demonstrating the signifcant influence of attitude on healthcare-seeking behaviour [38]. According to the TRA, such negative attitudes are likely to weaken behavioural intentions to use IPTp-SP. Together, these findings suggest that improving pregnant women's knowledge and addressing misconceptions about IPTp-SP may strengthen positive attitudes and, consequently, increase their intention to use the medication for malaria prevention.

Similar to an earlier study [39], the stakeholders in this study encouraged pregnant women to attend ANC and adhere to IPTp-SP regimens, mainly when prescribed by qualified HCPs. However, although supportive social influence was present, it was insufficient to motivate uptake among many pregnant women. While the TRA posits that subjective norms shape behavioural intentions, our findings suggest that supportive normative influences alone were not enough to promote IPTp-SP use. Despite encouragement from significant others, including community leaders, some pregnant women rejected IPTp-SP because their personal beliefs, misconceptions, and concerns about the medication outweighed these social influences. These findings highlight that the influence of subjective norms may be limited when individuals hold strong opposing attitudes or exercise personal agency in their decision-making. They also suggest that additional contextual and psychosocial factors, beyond those explicitly accounted for by the TRA, may influence uptake of IPTp-SP. Additionally, some pregnant women rejected advice from trusted individuals, indicating that normative influence did not always translate into behavioural change when it conflicted with women's own beliefs and perceptions of IPTp-SP. Together, these findings suggest that while community sensitisation efforts should engage influential stakeholders surrounding pregnant women, interventions must place pregnant women at the centre. Ultimately, pregnant women exercise agency in deciding whether to accept or decline IPTp-SP, highlighting the need for interventions that directly address their knowledge, attitudes, and concerns about the medication.

Although some pregnant women in this study expressed favourable attitudes and intentions toward IPTp-SP use, particularly when it was available and prescribed by trusted HCPs, uptake remained low. Although the TRA posits that an individual’s decision to adopt a particular behaviour is significantly dependent on their behavioural intention [24,26], it does not account for all determinants of IPTp-SP uptake, particularly structural factors. The gap between intention and behaviour observed in this study suggests that structural barriers, such as limited access to clinics, the unavailability of IPTp-SP, and inconsistent prescription of IPTp-SP by HCPs, may prevent women from translating positive intentions into actual use of IPTp-SP. These findings highlight the importance of considering structural constraints alongside psychosocial determinants when seeking to understand and improve IPTp-SP uptake.

Findings from this study suggest that the explanatory power of the TRA may be limited in contexts where structural and health-system barriers influence behaviour. According to the TRA, behaviour results from behavioural intentions, which are shaped by attitudes and perceived social norms. While many participants in this study held negative attitudes toward IPTp-SP use, those who expressed favourable intentions to use it still did not follow through with actual uptake. This disconnect between intention and behaviour could be attributed to structural barriers, such as inaccessible healthcare facilities and the unavailability of IPTp-SP, factors that the TRA does not account for. These findings highlight that behavioural theories such as the TRA, which assume the presence of enabling environments, may not apply uniformly across settings. Therefore, such theories should be applied with caution and contextual sensitivity, particularly in resource-constrained environments.

### Limitations

FGDs and IDIs as data collection methods allowed for a comprehensive exploration of participants’ experiences and perceptions, tapping into the richness and depth of their everyday lives. Conducting interviews in Pidgin English, Ijaw, and English based on participants’ linguistic preferences ensured effective communication and understanding. This study, however, had several limitations. First, these data were collected in 2022; however, the findings remain relevant because many of the behavioural and structural barriers to IPTp-SP uptake identified in this study continue to be documented in recent literature. Nevertheless, ongoing changes in malaria prevention policies and programme implementation should be considered when interpreting the findings. Second, although bilingual research assistants who were indigenous to the study communities interpreted the interview questions and translated the interviews into English, the absence of a formally translated interview guide may have resulted in minor variations in the wording or interpretation of some questions. This could have influenced participants' responses and should be considered when interpreting the findings. Third, information on participants' ANC attendance was not collected. As IPTp-SP is delivered through routine ANC services, the absence of these data limited our ability to examine how ANC utilisation influenced uptake of IPTp-SP among participants. Finally, convenience sampling through TBAs was used to recruit participants. While this facilitated access to information-rich participants in the studied communities, it may have introduced selection bias, as these women may differ from those who do not seek healthcare and utilise ANC. This may limit the transferability of findings and could have influenced the observed patterns of IPTp-SP uptake.

## Conclusions

According to the TRA, behaviour is driven by behavioural intentions, which are shaped by attitudes toward the behaviour and perceived social norms. In this study, attitudes toward uptake of IPTp-SP were generally poor, largely due to limited knowledge of its efficacy and safety. Although participants expressed social support and intention to use IPTp-SP, these did not necessarily translate into actual use. This gap is closely tied to structural barriers, including limited access to healthcare facilities and the unavailability of IPTp-SP. To improve IPTp-SP uptake, targeted educational campaigns are needed to correct misconceptions about the drug. Additionally, efforts should be made to ensure consistent availability and accessibility of healthcare centres and IPTp-SP, particularly in rural areas. Although this study focused on psychosocial determinants of IPTp-SP uptake, ANC attendance remains a critical upstream determinant of access to IPTp-SP. Future research should examine the factors influencing ANC attendance in rural Bayelsa communities to provide a more comprehensive understanding of barriers to IPTp-SP uptake and to identify opportunities to improve malaria prevention during pregnancy. .
